# What Snow Water May Hold

**Published:** 1874-04

**Authors:** 


### WHAT SNOW WATER MAY HOLD.

THE *Montreal Star* says that Dr. Edwards has addressed a communication to the Mayor of that city on the subject of the refuse covered up by the snow on the streets, which eventually, he thinks, will be desiccated by March winds, distributed as fine dust in the houses, and inhaled in the streets. He says that he finds that within an hour or two of melting the snow water contains a swarm of living organisms, including most of the infusory animalcules and a variety of worms and vibriones, a teaspoonful becoming, in fact, a miniature aquarium, and a few grains of the dust mentioned containing more filth, animal life, and germs of disease than a block of ice 600 pounds in weight. Dr. Edwards urges the Board of Health of the city to remove the unwholesome nuisance from the streets more promptly and efficiently than in former years, and represents the danger to health from deposits in hose cisterns, which he found in every case teeming with active animal life. He also addressed an argument to the Chairman of the Water Committee, maintaining that the filtration of the water supply, which would be of great economic value, may be effected at a moderate outlay. He mentions the deposits from the Ottawa and St. Lawrence waters, and states that water is filtered at Liverpool at the rate of about £575 per annum for each 1,000,000 gallons per day. He believes that the adoption of the Liverpool district plan in Montreal, of which filtration is the first element, would first double the available supply; secondly, afford also a spare head of water for flushing and cleansing streets; thirdly, improve the sanitary condition of the city by the supply of filtered water, and thus guard against prevailing endemic and threatened epidemic diseases, reduce the rate of infant mortality, and promote the general health and sobriety of the citizens at large.

